# Schizophrenia and Category-Selectivity in the Brain: Normal for Faces but Abnormal for Houses

**DOI:** 10.3389/fpsyt.2018.00047

**Published:** 2018-02-23

**Authors:** Lisa Kronbichler, Renate Stelzig-Schöler, Brandy-Gale Pearce, Melanie Tschernegg, Sarah Said-Yürekli, Luise Antonia Reich, Stefanie Weber, Wolfgang Aichhorn, Martin Kronbichler

**Affiliations:** ^1^Centre for Cognitive Neuroscience and Department of Psychology, University of Salzburg, Salzburg, Austria; ^2^Neuroscience Institute, Christian-Doppler Medical Centre, Paracelsus Medical University, Salzburg, Austria; ^3^Department of Psychiatry, Psychotherapy and Psychosomatics, Christian-Doppler Medical Centre, Paracelsus Medical University, Salzburg, Austria; ^4^Department of Psychiatry and Psychotherapy, University Medical Center Hamburg-Eppendorf, Hamburg, Germany

**Keywords:** schizophrenia, face perception, scene perception, fMRI, neuroimaging, specialisation

## Abstract

Face processing is regularly found to be impaired in schizophrenia (SZ), thus suggesting that social malfunctioning might be caused by dysfunctional face processing. Most studies focused on emotional face processes, whereas non-emotional face processing received less attention. While current reports on abnormal face processing in SZ are mixed, examinations of non-emotional face processing compared to adequate control stimuli may clarify whether SZ is characterized by a face-processing deficit. Patients with SZ (*n* = 28) and healthy controls (*n* = 30) engaged in an fMRI scan where images of non-emotional faces and houses were presented. A simple inverted-picture detection task warranted the participants’ attention. Region of interest (ROI) analyses were conducted on face-sensitive regions including the fusiform face area, the occipital face area, and the superior temporal sulcus. Scene-sensitivity was assessed in the parahippocampal place area (PPA) and served as control condition. Patients did not show aberrant face-related neural processes in face-sensitive regions. This finding was also evident when analyses were done on individually defined ROIs or on in-house-localizer ROIs. Patients revealed a decreased specificity toward house stimuli as reflected in decreased neural response toward houses in the PPA. Again, this result was supported by supplementary analyses. Neural activation toward neutral faces was not found to be impaired in SZ, therefore speaking against an overall face-processing deficit. Aberrant activation in scene-sensitive PPA is also found in assessments of memory processes in SZ. It is up to future studies to show how impairments in PPA relate to functional outcome in SZ.

## Introduction

Schizophrenia (SZ) is a complex psychiatric disease characterized by positive and negative symptoms, cognitive deficits, and a severe impairment of social cognitive functions (1–5). A possible explanation for aberrant social abilities may be general impairments in the processing of facial information (6, 7). SZ patients show difficulties during a variety of face tasks revealing aberrant face detection abilities as well as slowed response times (8–11). SZ is also related to aberrant eye movements and fixations during face perception (12), but less during the processing of other stimulus categories (13).

Faces are a unique class of visual objects which are processed in a specialized set of cortical areas. The fusiform face area, which is located at the lateral side of the mid-fusiform gyrus, is one of the most prominent regions associated with face processing [FFA (14)]. Additional face-responsive regions in the extrastriate cortex are located in the inferior occipital gyrus (OFA) and the superior temporal sulcus (STS), which are related to early face processing and the perception of gaze and emotions, respectively (6).

Critically, Quintana et al. (15) found no evidence for right FFA activation during face processing in SZ patients, whereas activation in the same region was evident in healthy controls. Another study (16) found that FFA activation in healthy participants was higher when faces were successfully recognized, whereas neural activation in patients was similar for all face types.

The finding of abnormal neural activation in the FFA in SZ is confirmed in a number of neuroimaging assessments (17–20), and structural investigations reveal remarkable gray matter reductions in the fusiform gyrus in patients (21–23). Critically, findings in this field are inconsistent, since several studies do not replicate abnormal FFA activation in SZ (24, 25).

A closer examination of the experimental designs used in the assessment of face processing in SZ shows that previously used setups strongly vary in task demands, which in turn might explain discrepant findings. Some studies used recognition tasks, which rely on working memory processes that are known to be impaired in SZ (26). Accordingly, such tasks might reflect memory impairments rather than face processing deficits *per se* (8, 16, 27). Additionally, emotional stimuli are frequently used, even in tasks designed to assess aspects of non-emotional face processing like gender or age decisions (25, 28, 29). This is insofar relevant, as emotional faces reveal quantitatively different response as compared to neutral faces. Eye movement studies suggest that SZ patients show abnormal eye movements during emotional face perception and even avoidance of certain facial features (30, 31). On the neuronal level, emotional faces revealed increased neural response in face-sensitive cortical areas and the limbic system (32). Other studies revealed that SZ patients show hyperactivation during non-emotional face perception, whereas emotional faces were associated with hypoactivation (33). This hypoactivation strongly resembled neural activation of patients during a Theory of Mind task assessed in the same study (34). Several current face-processing studies presented emotional stimuli, thus prompting the question whether the identified impairments represent true face-processing deficits or reflect confounding effects of impaired social cognition. In addition, as recently pointed out by Maher et al. (35), face perception includes not only face-specific processing but also more general perceptual processes. Therefore, to distinguish between pure face-specific processing impairments and general processing impairments, the assessment of cortical activation during control stimuli other than faces is inevitable (25). Maher et al. (35) could show that face-processing deficits in SZ might not only become evident *via* diminished FFA activation as a whole but may rather be characterized by a diminished contrast between face and other object stimuli in this region. Also, investigations on processing impairments of higher-order visual areas are restricted to face-selective areas and literature on other category-specific regions [e.g., extrastriate body area, parahippocampal place area (PPA)] is sparse.

The PPA, for example, is specialized for the processing of visual information about scenes and spatial navigation (36, 37). Studies assessing PPA activation make use of the region’s specific response toward scenes (38) and buildings (39). The investigation of other category-specific regions like the PPA in the frame of SZ research is insofar relevant, as the finding of similarly impaired specialization in other higher-order visual areas would draw a completely different picture; Decreased specialization for stimulus classes beside faces would indicate that there is a rather general malfunction in higher visual cortex.

In the current study, we acquired the blood oxygen level-dependent (BOLD) response of SZ patients and healthy control participants during the processing of non-emotional faces. To evade confounding effects of memory impairments, participants had to detect inverted target stimuli. Thus, successful task performance did not require memory processes. Pictures of houses were used as control stimuli in order to quantify the magnitude of face sensitivity in the FFA and house-sensitivity of the PPA. Our assumptions were twofold: given that there is a distinct face-processing deficit in face-selective regions in SZ, we should find altered neural response in SZ patients in the FFA during face processing. This effect might as well be reflected in a quantitative difference in the face vs. house contrast between groups (35). Second, if stimulus-sensitivity is exclusively impaired during face processing, we should not be able to identify deficient house-specific activation in the house-sensitive PPA. If we identify aberrant response toward house stimuli, this would challenge the assumption of a deficit in visual processing that is restricted to facial stimuli. We investigate neural response in four predefined regions of interest (ROIs), namely, bilateral FFA and bilateral PPA. Investigations on additional face-sensitive regions like occipital face area and STS are provided. To account for the drawback of using healthy-participant group ROIs on patients, we did additional analyses on individually defined ROIs of peak activation for face and house stimuli.

## Materials and Methods

### Participants

Participants in the patient group were 28 male adults, who had received a formal ICD-10 diagnosis (which was checked before study participation by certified psychiatrists) in the schizophrenia spectrum group (F20) or the schizoaffective disorders spectrum group (F25). All patients were recruited from the outpatient and inpatient units of the Department of Psychiatry, Psychotherapy and Psychosomatics. All patients received antipsychotic medication (mean chlorpromazine equivalent = 302.8). Patients were clinically stable with relatively mild symptoms at the time of fMRI assessment [PANSS (40)]. Only male patients and control participants were examined since patients were recruited from a Department (see above), which, at that time, predominantly housed male patients and it would not have been possible to recruit a sample balanced for male:female ratio. Healthy control participants were 31 male adults. Efforts were made to recruit a healthy male control group that matched the SZ group in demographics and education. Thus, advertisements for HCs specified that we were particularly interested in participants who finished high school, but did not necessarily attend or complete college. Exclusion criteria for both, patients and healthy controls, were psychiatric disorders other than SZ or schizoaffective disorders, fMRI incompatibility, current or past neurological insults like head trauma, and current substance abuse.

Controls were screened for mental and physical health (*via* a standardized anamnesis procedure) and were excluded if they reported a current or history of mental or neurological disorder or a family history of psychiatric disorders. Absence of mental disorders in controls was further checked by the Mini-International Neuropsychiatric Interview (41), performed by a trained psychiatrist or psychologist. To examine potential subclinical symptoms of SZ spectrum disorders, all controls completed the German version of the Schizotypal Personality Questionnaire (42). None of the controls scored more than 1.5 SD above the mean of reported norms in healthy subjects on that measure (42).

All participants were screened for cognitive impairments [SCIP (43)]. This scale is well suited for the assessment of cognitive impairments in psychiatric ill patients (44) and includes list learning, consonant trigrams, oral fluency, delayed list learning, and a visuomotor assignment task. Subjects were remunerated for participation and all participants provided written informed consent in accordance with the Declaration of Helsinki.

All methods conform to the Code of Ethics of the World Medical Association (Declaration of Helsinki). The institutional guidelines of the University of Salzburg (Statutes of the University of Salzburg—see https://online.uni-salzburg.at/plus_online/wbMitteilungsblaetter.display?pNr=98160) state in § 163 (1) that ethical approval is necessary for research on human subjects if it affects the physical or psychological integrity, the right for privacy or other important rights, or interests of the subjects or their dependents. In § 163 (2), it is stated that it is the responsibility of the PI to decide, whether (1) applies to a study or not. Data was processed in anonymized/deidentified form. Upon arrival at the lab, participants were assigned a subject ID (v001, v002, etc.), which was used throughout the study. Considering the patient sample, the study was part of a longitudinal study including behavioral and MRI acquisitions in affective and psychotic disorders approved by the local ethics committee (Ethikkommission für das Bundesland Salzburg). Demographic data and clinical rating are listed in Table 1. Education levels, handedness, and medication doses are provided in Tables S2 and S3 in Supplementary Material.

**Table 1:** Demographic data and clinical rating of schizophrenic patients and controls.

Group	*n*	Age (years)	Illness duration (years)^b^	SCIP^a^	PANSS+	PANSS−
Patient	28	25.85 (4.9)	3.9 (4.7)	69.67 (11.63)	14.12 (5.8)	15.56 (6.8)
Control	31	25.43 (4.3)		84.71 (7.4)		

*SD in parentheses; n, number of participants*.

*^a^Significant *t*-test (*p* < 0.05)*.

*^b^Illness onset is defined as timepoint of first professional help seeking*.

### Stimuli and Design

All house stimuli and several face stimuli were downloaded from the public domain of the World Wide Web. Additional face stimuli were taken from a standardized corpus downloaded from https://www.macbrain.org.^1^ All house and face stimuli were grayscale 1,024 × 768 pixel images, face and house stimuli were matched in size, luminance and contrast [SD of luminance see in Ref. (45)] to the pictures of the corpus. Note that there is an ongoing discussion on the contribution of other low-level image properties to category-selective response [e.g., Ref. (46, 47)] and future studies are needed to explore whether and to which extent differences in these low-level properties contribute to abnormal brain responses in higher visual cortex in SZ. All face stimuli showed a neutral emotional expression.

Stimuli were presented centrally on a black background on a MRI compatible LCD monitor and seen by participants *via* a mirror mounted on the head coil. Two consecutive scan sessions were conducted. In total, the participants attended 180 upright face and 180 upright house stimuli. Stimulus order was pseudo-randomized. Each stimulus was presented for 750 ms and stimuli were separated by a blank screen for a jittered time interval of 1,500–3,500 ms. To maintain their attention, participants had to indicate (*via* right thumb button press), when pictures were presented upside down. The experiment included 20 inverted house and 20 inverted face target trials. Target pictures were distributed randomly across the experiment and were modeled as factors of no interest in fMRI analyses. Behavioral results are described in the Supplementary Material.

### Image Acquisition and Data Analysis

Functional imaging data were acquired with a Siemens Magnetom Trio 3 T scanner (Siemens AG, Erlangen, Germany) using a 32-channel head coil. Functional images sensitive to BOLD contrast were acquired with a T2*-weighted gradient echo EPI sequence (TR 2,250 ms, TE 30 ms, matrix 64 mm × 64 mm, FOV 192 mm, flip angle 70°). Thirty-six slices with a slice thickness of 3 mm and a slice gap of 0.3 mm were acquired within the TR. Scanning proceeded in two sessions with 321 scans per session. Six dummy scans were acquired at the beginning of each functional run before stimulus presentation started. Additionally, a gradient echo field map (TR 488 ms, TE 1 = 4.49 ms, TE 2 = 6.95 ms) and a high-resolution (1 mm × 1 mm × 1 mm) structural scan with a T1-weighted MPRAGE sequence were acquired from each participant.

For preprocessing and statistical analysis, SPM12 software,^2^ running in a MATLAB R2013a environment (Mathworks Inc., Natick, MA, USA), and additional functions from AFNI^3^ were used. Functional images were realigned, de-spiked (with the AFNI 3ddespike function), unwarped, and corrected for geometric distortions using the fieldmap of each participant and slice time corrected. The high resolution structural T1-weighted image of each participant was processed and normalized with the CAT12 toolbox^4^ using default settings, each structural image was segmented into gray matter, white matter and CSF and denoised, then each image was warped into MNI space by registering it to the DARTEL template provided by the CAT12 toolbox *via* the high-dimensional DARTEL (48) registration algorithm. Based on these steps, a skull stripped version of each image in native space was created.

To normalize functional images into MNI space, the functional images were coregistered to the skull stripped structural image and the parameters from the DARTEL registration were used to warp the functional images, which were resampled to 3 mm × 3 mm × 3 mm voxels and smoothed with a 6 mm FWHM Gaussian kernel.

Statistical analysis was performed with a general linear model (GLM) two-staged mixed effects model. In the subject-specific first level model, each condition was modeled by convolving stick functions at its onsets with SPM12’s canonical hemodynamic response function [target trials and start and end messages were modeled as separate events of no interest, the model also included the six motion parameters and six noise regressors, reflecting physiological noise components obtained from FIACH (49) as regressors of no interest]. Parameter estimates for each condition were calculated *via* these first-level GLMs, using a temporal high-pass filter (cutoff 128 s) to remove low-frequency drifts and modeling temporal autocorrelation across scans with an AR (1) process (50).

For voxel-based group analyses, contrast images for effects of interest were calculated at the first level and rescaled to increase statistical sensitivity and decrease interindividual variability by the Vascular auto-rescaling of fMRI (VasA fMRI) technique (51). These rescaled contrast images were used in second level analyses for a 2 (stimulus type: faces and houses) × 2 (group: SZ and controls) ANOVA implemented *via* one-way ANOVAs at the second level for the main effects of stimulus, group, and the interaction. All results from whole brain analyses are reported at a voxel-level threshold of *p* < 0.001 (uncorrected) with a FWE cluster-level correction of *p* < 0.05.

### ROI Analyses

For the ROI analyses, we extracted contrast estimates from the *faces* > *baseline* and *houses* > *baseline* first-level rescaled contrast image of each participant averaging across all voxels within the left FFA (*x* = −36; *y* = −49; *z* = −17; ext = 22), right FFA (*x* = 36; *y* = −49; *z* = −17; ext = 68), left PPA (*x* = −27; *y* = −64; *z* = −11; ext = 433), and right PPA (*x* = 30; *y* = −52; *z* = −8; ext = 409) ROIs. ROIs were based on the maximum-probability maps from the freely available probabilistic Brain Activity Atlas [BAA (52, 53)]. For the FFA ROIs, we combined the anterior and posterior FFA ROIs from the BAA into combined FFA ROIs (one for each hemisphere). Details are described in the Supplementary Material. Subsequent analyses were done using IBM SPSS Statistics 20^®^. The results of the group ROIS are presented in the Section “Results.” We conducted 2 × 2 factorial ANOVAs with the factors stimulus type (face/house) and group (patients/controls). For the sake of comparison, we did additional analyses on individually defined ROIS which are described in the Supplementary Material. Box-plots were design with an R-based web tool described in the study by Spitzer et al. (54).

For our analyses on in-house ROIs, FFA and PPA cluster were defined on an in-house fMRI data-set. FMRI scans were obtained from 40 healthy participants viewing visually presented face and house stimuli. Data were acquired with the same Siemens Magnetom Trio 3 T scanner.

## Results

### ROI Analyses

Both FFA ROIs of the BAA revealed significantly higher activation for face compared to house stimuli [*F*s(1,57) > 47.45, *p*s < 0.001]. This main effect for stimulus was independent of group since no stimulus-by-group interaction could be observed [*F*s(1,57) < 2.42, *p*s > 0.12]. A main effect of group was marginally significant in the left anterior–posterior FFA [*F*(1,57) = 3.9, *p* = 0.051]. Here, SZ patients revealed overall decreased neural response compared to healthy controls. Additional analyses show that effects did not systematically vary with hemisphere (see Supplementary Material).

Left and right PPA ROI of the BAA showed significantly higher activation for house compared to face stimuli [*F*s(1,57) > 301, *p*s < 0.001]. Patients revealed decreased overall activation in bilateral PPA [*F*s(1,57) > 4.69, *p*s < 0.034]. Notably, these main effect were qualified by a significant stimulus-by-group interaction in left and right PPA [*F*s(1,57) > 14.4, *p*s < 0.001].*Post hoc t*-tests revealed significantly lower neural response of SZ patients (compared to controls) toward house stimuli (*t*s > 3.25, *p*s < 0.002) but similar neural response for face stimuli (*t*s < 1.17, *p*s > 0.24) in both PPA clusters.

**Figure 1:**
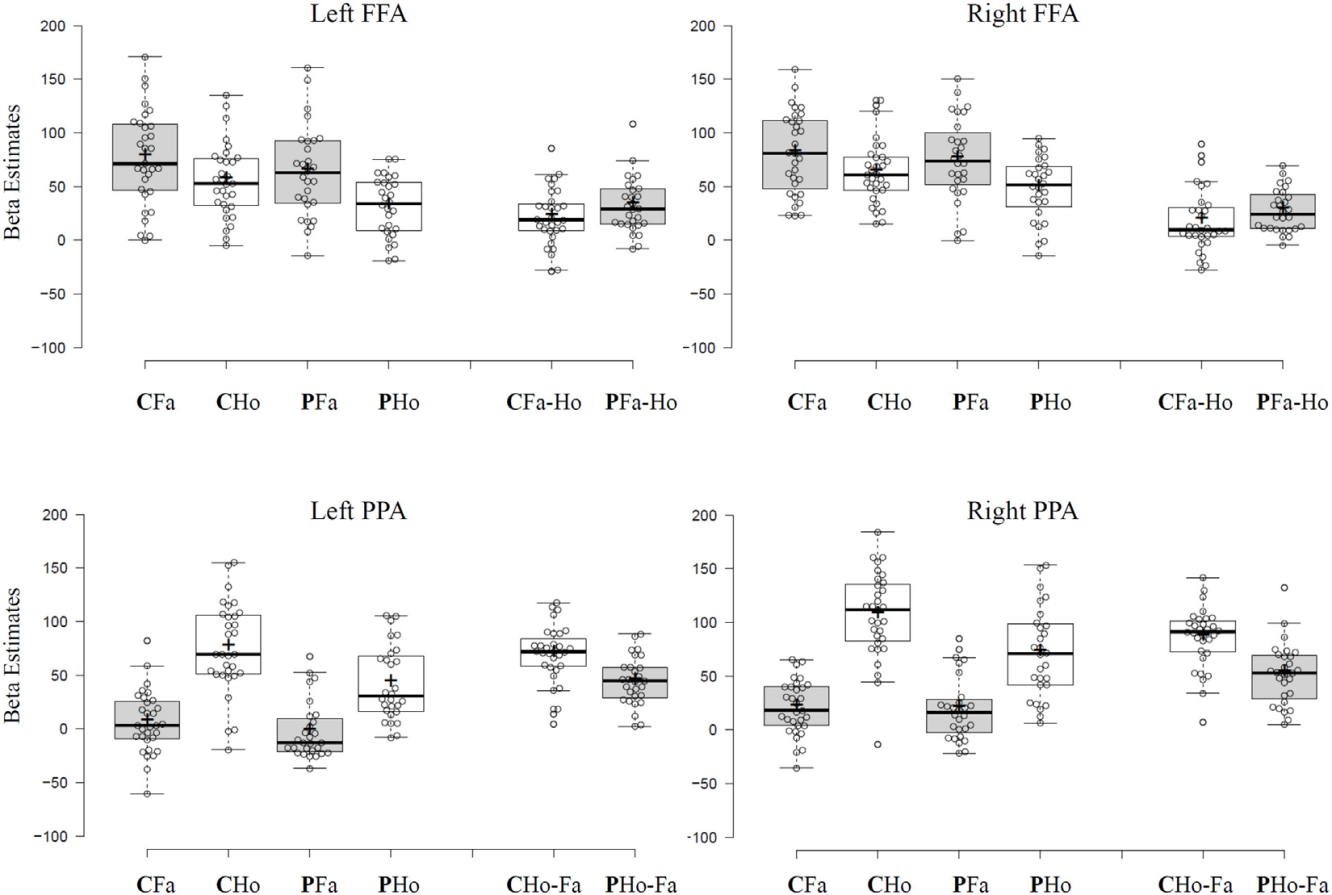
Tukey box-plots depict beta estimates extracted from left and right FFA and parahippocampal place area (PPA) group regions of interest from the Brain Activity Atlas. Bold horizontal lines indicate the group median, bold crosses show the group mean. End of whiskers indicate the first and third quartile. Abbreviations: C, control participants; P, schizophrenic patients; Fa, face stimuli; Ho, house stimuli; *Fa-Ho* shows differential scores calculated by subtracting mean subject beta estimates for house stimuli from mean subject beta estimates for face stimuli in *Faces* > *Houses contrast* cluster. *Ho-Fa* shows differential scores calculated by subtracting mean subject beta estimates for face stimuli from mean subject beta estimates for house stimuli in *Houses* > *Faces contrast* cluster.

Taken together, SZ patients showed altered house-specific activation in house-sensitive PPA, whereas no significant alterations were identified for face stimuli (results are illustrated in Figure 1). To account for the possibility that the PPA might be differently positioned in SZ and that therefore our group PPA ROIs might mislocate the actual PPA in patients, we did additional analyses where we searched for house-sensitive and face-sensitive clusters in each participant individually. In an alternative approach, we used PPA and FFA ROIs from an in-house localizer task to validate our findings.

Remarkably, the critical finding remained the same irrespective of ROI positioning method used: patients showed decreased house-specific activation in left and right PPA, whereas there was no observable difference in face-related activation between groups in both PPA and FFA ROIs. Additional analyses on other face-sensitive regions like occipital face area (OFA) and STS also revealed no evidence for aberrant face sensitivity in our patient sample. All *F* values, a detailed description, and figures of these additional analyses are available in the Supplementary Material.

Additionally, we assessed whether face- and house selectivity in the FFA and PPA, respectively, varied systematically with clinical, cognitive, or social functioning measures collected within the frame of this study. House-sensitivity was found to vary with positive and negative symptoms, that is, decreased house-sensitivity was related to stronger positive and negative symptoms. However, these analyses were not hypothesis driven and must be seen as additional exploratory analyses. Their findings are reported in Table S4 in Supplementary Material.

### Whole Brain Analysis

**Figure 2:**
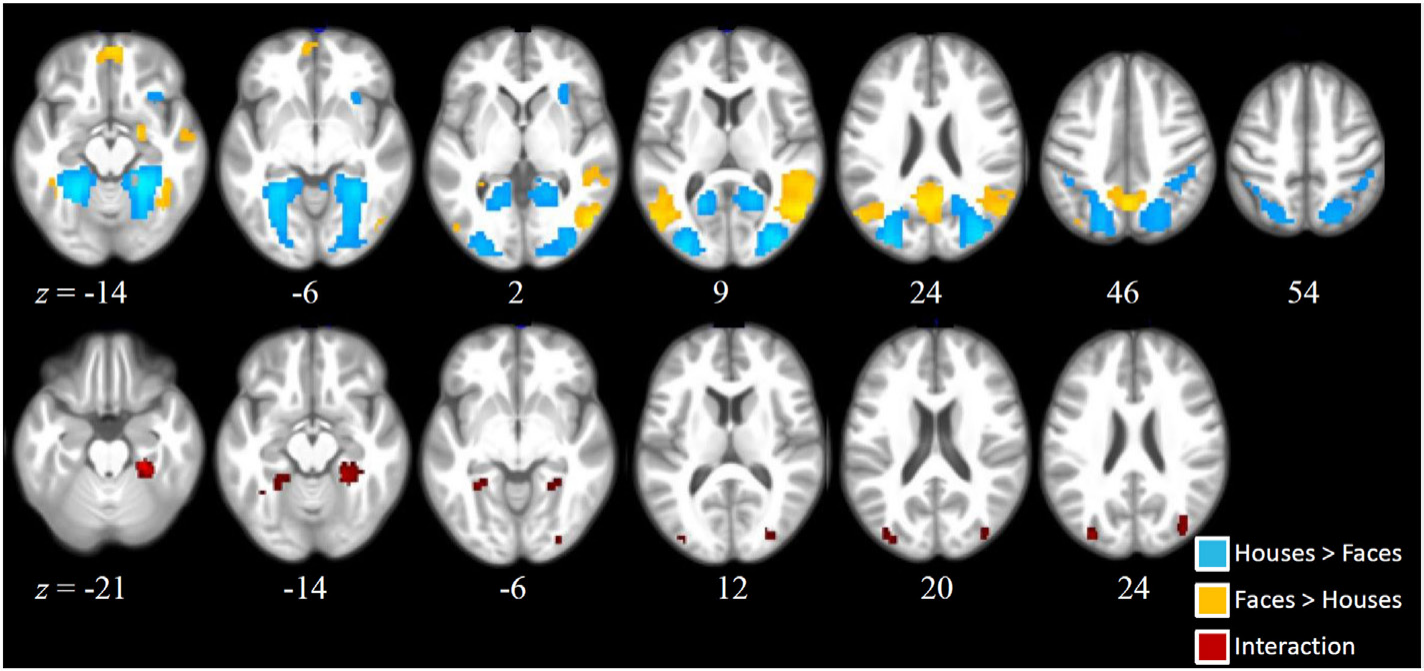
Activation clusters revealed by the whole brain analyses. Regions that elicited increased activation for *houses* compared to *faces* (irrespective of group) are illustrated in blue. Regions that elicited increased activation for *faces* compared to *houses* (irrespective of group) are shown in yellow. Red spots mark clusters where stimulus effects (faces vs. houses) were modulated by group (i.e., interaction). All clusters were extracted at a threshold of *p* < 0.001 [uncorrected, with a FWE cluster-level correction (*p* < 0.05)].

As can be seen from Figure 2, main effects of stimulus (across both groups) were found in a number of brain regions. Higher activation for faces compared to houses (shown in warm colors) were found in a number of regions commonly involved in face processing, including a right and a left fusiform cluster, corresponding to the FFA, but also in an extended medial parietal cluster, in a medial frontal cluster and in extended clusters in bilateral temporoparietal cortex (extending from the angular gyrus to middle temporal and STS regions) and in the right amygdala (see Table 2).

**Table 2:** Significant cluster of the whole brain analysis.

	MNI coordinates			Volume (voxels)	
Region	*x*	*y*	*z*		*F*
**Stimulus main effect**					
*Face* > *House*					
Medial parietal	3	−58	43	493	74.71
Right temporoparietal	57	−61	16	653	68.65
Right fusiform	39	−52	−17	32	66.00
Right amygdala	18	−7	−17	30	63.94
Left temporoparietal	−54	−64	15	294	46.27
Medial frontal	3	56	−14	91	40.81
Right anterior middle temporal	57	−4	−20	49	30.51
Left fusiform	−36	−49	−17	6	18.20
*House* > *Face*					
Right medial occipitotemporal and posterior occipital				1,709	
Lingual	24	−43	−11	–	405.61
Middle occipital	33	−82	10	–	249.86
Parahippocampal	27	−31	−20	–	233.89
Left medial occipitotemporal and posterior occipital				1,440	
Lingual	−21	−46	−11	–	321.79
Middle occipital	−30	−85	19	–	289.72
Occipital fusiform	−27	−61	−11	–	227.51
Right supramarginal	48	−34	49	97	33.16
Right anterior insula	30	32	1	72	24.86

Stimulus*group interaction					
Right parahippocampal	27	−34	−20	110	33.83
Left parahippocampal	−24	−40	11	63	29.84
Left middle/superior occipital	−30	−82	25	37	19.83
Right middle/superior occipital	39	−82	19	57	18.05

*Data were extracted at a voxel-level threshold of *p* < 0.001 (uncorrected) and a cluster-level threshold (FWE) of *p* < 0.05*.

House stimuli led to higher activation in a left and a right extended medial occipitotemporal and occipital cluster, including parahippocampal, lingual, medial occipito-parietal, and posterior middle and superior occipital regions, closely corresponding to previously identified scene-selective regions [PPA, retrosplenial cortex, and transverse occipital sulcus (TOS)]. Two smaller clusters with higher activation for houses than faces were found in the right supramarginal gyrus and in the right anterior insula.

**Figure 3:**
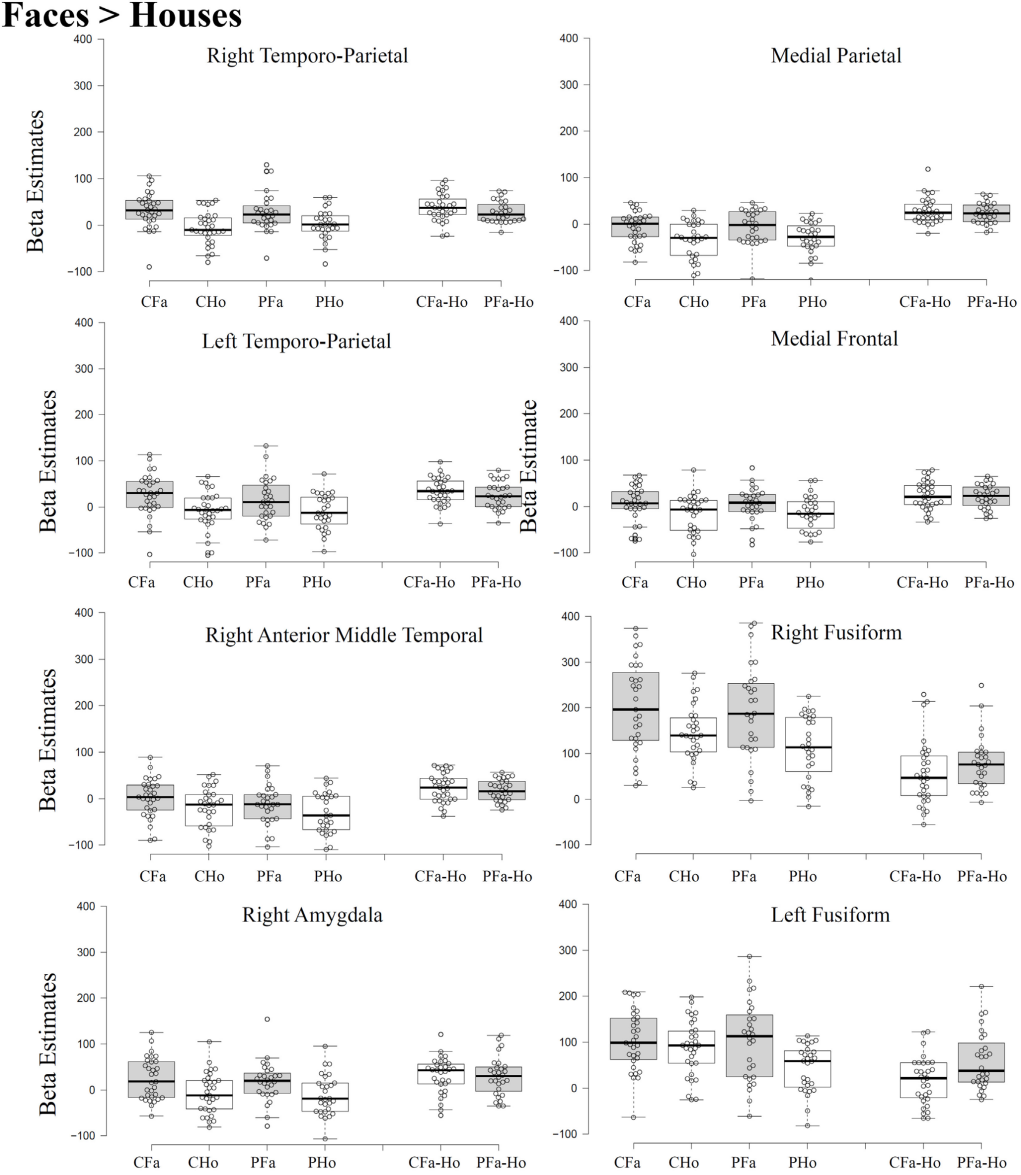

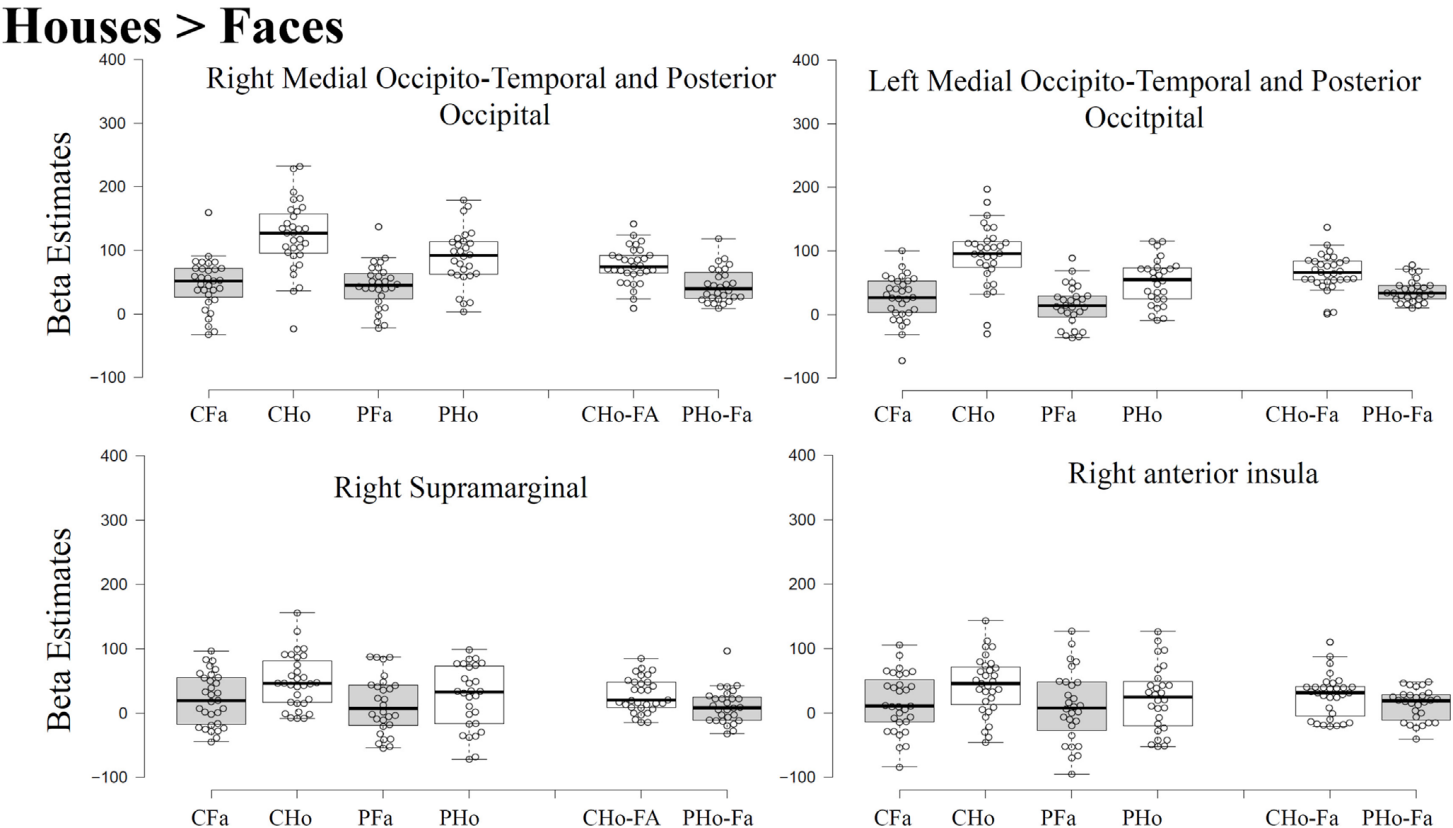
Beta estimates extracted from significant cluster of the whole brain analysis. Data were extracted at a threshold of *p* < 0.001 [uncorrected, with a FWE cluster-level correction (*p* < 0.05)]. For Tukey box-plot descriptions, see caption of Figure 1.

Statistically significant interactions between group and stimulus (see Table 1) were found in a left and in a right parahippocampal cluster (extending into anterior lingual and medial occipitotemporal gyri) closely corresponding to the PPA and in a left and a right occipital cluster, including middle and superior occipital regions, close to the previously described scene-selective TOS regions. As can be seen from the signal change plots for these four clusters in Figure 3, in each of these clusters patients with SZ showed decreased selectivity for houses vs. faces compared to the control group, mainly due to decreased activation for houses in patients, whereas face stimuli evoked nearly the same activation in these house-selective regions in both groups. For comparison, signal change plots for those clusters showing a main effect of stimulus, but not stimulus-by-group interaction (see Figure 3) show that face-selective regions respond with a similar profile and magnitude in both groups. No clusters with a significant main effect of group were identified at the chosen threshold.

## Discussion

Past studies on face processing in SZ revealed mixed results with some supporting a face-processing deficit in SZ, whereas others do not (6, 7). In the current study, we presented non-emotional faces and control house stimuli during a simple target detection task which did not rely on working memory. Participants responded to inverted face and house stimuli and overall hit rate was adequate for both groups (>74%). Although inverse face detection might require less processing depth as compared to, for example, face recognition or gender judgments, this task robustly identifies face-sensitive cortical areas like FFA, OFA, and STS in numerous studies [(55) for a review, see Ref. (56)]. Furthermore, these regions reveal solid face-specific activation even during passive viewing (56).

To our knowledge, this is the first study to compare the extent of stimulus specialization in the FFA (and OFA/STS, see Supplementary Material) to stimulus specialization in another area of the ventral visual stream, namely the PPA (36). Strikingly, we were not able to replicate deficient face-processing deficits but rather support findings of normal face-specific activation in SZ (24, 25). Patients’ neural response toward face stimuli in the FFA did not differ from healthy controls. To illustrate, both, faces contrasted against baseline activation (empty screen) and faces contrasted against houses (thus showing the amount of truly *face*-sensitive activation) was comparable between patients with SZ and healthy controls. Together with other studies, this finding challenges the notion of deficient face processing in SZ—at least for non-emotional stimuli. To account for the possibility that patients’, FFA was mislocated by our use of a group ROI, we did additional analyses in individually defined FFA. Peak coordinates of the groups were similar (57) and never more than 3 mm apart (see Table S1 in Supplementary Material), therefore indicating a similar location of the FFA in both groups. Results of the individual FFA ROIs were in accordance with our group ROIS since, again, no face-deficit could be observed. OFA and STS, two face-sensitive regions (32, 58) largely neglected in previous face-processing research in SZ, also did not reveal any evidence for aberrant face-related processes. Notably, there actually was a stimulus-by-group interaction in left OFA, but this was caused by decreased *house*-related neural activation in patients. Other ROI analyses based on an in-house functional localizer, again, could not identify face-related deficits in patients.

As we mentioned in the Section “Introduction,” the existing studies on face processing vary with respect to task, memory load and clinical factors and the attempt to integrate our findings in the available pool of studies (and drawing conclusions from our findings) must be done with great caution. To illustrate, we used a task that requires relatively low working memory load and revealed no abnormalities in schizophrenic face processing. This is in line with current research by Anilkumar et al. (24), whose task required minimal memory processes and who also attested normal face-related neural response in the FFA. Critically, other studies (again with low memory load) *do* show abnormal FFA activation in SZ (15, 29). Therefore, it seems like working memory load alone is not a reliable predictor of FFA abnormalities. With respect to the clinical characteristics of the schizophrenic sample, our patients have an illness duration of approximately 3.9 years, and are, on average, 26 years old. Together with previous findings on first-episode patients (24), one could assume that due to the relatively short illness duration and the young age, our participants’ neural activation is more healthy-like compared to elder patients with a longer illness duration [for example, Ref. (29)]. However, young participant groups are also examined in studies that actually show aberrant face processing (16) and there are studies on elder patients who do not reveal processing abnormalities (25). As evident from the participants’ description, our sample exclusively consisted of male patients. Although there is evidence that social cognition in healthy participants varies with sex (59), we are not aware of studies showing that overall FFA (or PPA) activation varies with sex. Predominantly male samples are involved in most studies on face perception in SZ (15, 16, 18, 29) which facilitates a comparison between our and previous studies. An exemplary exception is the study by Anilkumar et al. (24), assessing seven male and six female medication-free patients. Interestingly, they did not show abnormal facial processing in the patient sample, which is in line with our findings. Either way, future studies are needed to evaluate on possible differences between male and female samples with respect to neuronal face processing, since, strictly spoken, the current findings are restricted to male patients. Another often discussed factor is participants’ medication. In our sample, antipsychotic medication was not related to neural activation in FFA and PPA (*r*s < 0.06, *p*s > 0.7) and behavioral face perception seems not to improve with antipsychotic medication (60). However, larger-scale studies on this topic are sparse. A majority of patients assessed in published studies is on antipsychotic medication [e.g., Ref. (15, 18, 25)], whereas some are not (24). However, visual inspection does not reveal an obvious relation between medication and neural face processing. Furthermore, the unsystematic way in which medication is reported makes it difficult to compare medication levels across studies. It will be the mammoth task of future studies to systematically meta-analyze single studies and to identify the factors that lead to (ab)normal FFA activation in SZ samples.

In stark contrast to the essentially normal activation for faces, we identified aberrant house-related activation in patients in the PPA (and more generally decreased specialization for house stimuli in scene-selective visual regions in the voxel-based analysis). Here, patients showed decreased house preference as evident in a decreased house vs. face differentiation. Notably, this effect was not caused by a deviant neural response toward faces, since face-related activation in PPA did not differ between patients and controls. Rather, patients showed decreased neural response to house stimuli in bilateral PPA. Again, we tested whether this effect arose from a mislocation of the PPA by our group ROIs. Individually selected PPA cluster did not differ in peak coordinates between groups (differences < 2 mm; see Table S1 in Supplementary Material). However, patients’ PPA cluster were significantly smaller compared to those identified in healthy controls, which is in line with the decreased scene-selectivity in patients in visual regions (see Figure 2).

The findings of our individually defined ROIs (and additional analyses computed on in-house PPA ROIs) revealed a robust pattern that supported our initial finding of decreased house-related activation in PPA in schizophrenic patients. To date, this is the first study revealing a specialization deficit in category-selective ventral visual cortex beyond the commonly assessed FFA. Notably, patients did not *lack* house-specialization, rather, the sensitivity of the PPA for house stimuli over other stimulus types was strongly reduced.

At first sight, aberrant visual specialization in a higher visual region of the ventral stream might be associated with deficient visual processing in early areas (61). If decreased, scene selectivity was caused by disturbed early visual processing, we might expect that also face-selectivity is impaired in schizophrenic patients and that we find group differences for all visual stimuli already in early visual regions. However, none of these assumptions were confirmed in the present study. Therefore, early visual deficits can hardly account for the pattern of deficient activation identified in our study—although low-level visual processing deficits are clearly present in SZ as documented in numerous previous studies (62–64). It is also possible that there are specific low-level visual features, which characterize scene stimuli vs. face stimuli and which in turn might explain parts of the scene-selective vs. face-selective activations in ventral visual cortex (65). Interestingly, eye-tracking studies found that SZ patients reveal aberrant visual scan paths during both, face as well as scene processing: besides overall decreased scanpath length, patients show less fixations (but longer fixation durations) and longer saccade amplitude (66). Further studies will be needed to show how abnormal visual scanpath relates to aberrant neural response in category-sensitive regions (e.g., *via* simultaneous eye-tracking and fMRI examinations) and whether such abnormalities can be explained by low-level visual features.

Aberrant parahippocampal activation was also identified in studies on SZ assessing declarative memory processes for visual stimuli (67, 68). Schizophrenic patients and healthy siblings engaged in an encoding and subsequent retrieval block depicting complex scenes. Both groups revealed diminished scene-specific activation that varied systematically with visual memory scores (68). Furthermore, schizophrenic patients are found to have impairments in correctly navigating through virtual landscapes (69, 70), an ability which relies on PPA processes (71). Ledoux et al. (72) even proposed that gray matter volume in this cortical area is positively related to successful navigation in SZ and control participants. Dysconnectivity between cortical regions might be a plausible explanation for distributed deficits in occipital cortex identified in SZ (73). Note that, although we did not find aberrant activation besides the PPA and scene-selective ventral visual cortex, we do not claim that schizophrenic patients exclusively suffer from PPA deficits. Rather, we aim to point out that deficits in visual processing in SZ go beyond FFA abnormalities. To illustrate, Surguladze et al. (74) revealed increased PPA activation in SZ patients while processing neutral faces. Abnormal neural response in this area was positively associated with reality distortion in the patient sample. Comparably, we could show that PPA activation abnormalities increased with increasing positive and negative symptoms.

Other interesting findings come from assessments on old adults who, similar to our SZ sample, show less differentiated neural response toward face and place stimuli (75, 76). Besides others, one theory here is that dedifferentiation might be caused by altered global connectivity, which is frequently found in old adults (77). It remains up to future studies to show how impaired category-sensitivity might be related to aberrant connectivity in SZ as well as in other (clinical) samples.

One should also note that our study has several limitations. The sample size, although larger compared to most previous studies on fusiform face area activation in SZ, is still relatively small and our findings should be confirmed by larger studies. Such future studies should also include patients without antipsychotic medication, as in our study, all patients received antipsychotic medications and we cannot exclude that the presently found abnormalities in scene-selective brain activations are influenced by medication (but note that previous studies found parahippocampal activation abnormalities during memory encoding also in non-medicated relatives of schizophrenic patients) (67, 68).

## Ethics Statement

All participants provided written informed consent in accordance with the Declaration of Helsinki. All methods conform to the Code of Ethics of the World Medical Association (Declaration of Helsinki). The institutional guidelines of the University of Salzburg (Statutes of the University of Salzburg—see https://online.uni-salzburg.at/plus_online/wbMitteilungsblaetter.display?pNr=98160) state in § 163 (1) that ethical approval is necessary for research on human subjects if it affects the physical or psychological integrity, the right for privacy or other important rights or interests of the subjects or their dependents. In § 163 (2), it is stated that it is the responsibility of the PI to decide, whether (1) applies to a study or not. Data was processed in anonymized/deidentified form. Upon arrival at the lab, participants were assigned a subject ID (v001, v002, etc.) which was used throughout the study. Considering the patient sample, the study was part of a longitudinal study including behavioral and MRI acquisitions in affective and psychotic disorders approved by the local ethics committee (Ethikkommission für das Bundesland Salzburg).

## Author Contributions

MK and WA designed the study. MT wrote the protocol. LK, MK, and MT managed the literature searches and analyses. RS-S managed and determined the psychiatric assessments. RS-S, B-GP, LR, and SW conducted the psychiatric assessments. LK and MT collected the fMRI data. SS-Y designed the scripts for statistical analyses of fMRI data. SS-Y and LK undertook the statistical analysis, and author LK and MK wrote the first draft of the manuscript. All authors contributed to and have approved the final manuscript.

## Conflict of Interest Statement

The authors declare that the research was conducted in the absence of any commercial or financial relationships that could be construed as a potential conflict of interest.

## Footnotes

^1^Development of the MacBrain Face Stimulus Set was overseen by Nim Tottenham and supported by the John D. and Catherine T. MacArthur Foundation Research Network on Early Experience and Brain Development. Please contact Nim Tottenham at tott0006@tc.umn.edu for more information concerning the stimulus set.

^2^http://www.fil.ion.ucl.ac.uk/spm/.

^3^https://afni.nimh.nih.gov/.

^4^http://dbm.neuro.uni-jena.de/cat.
