# Supplementary material for: Schizophrenia and Category-Selectivity in the Brain: Normal for Faces but Abnormal for Houses
Source: Front Psychiatry. 2018 Feb 23;9:47. doi: 10.3389/fpsyt.2018.00047 (PMC5829027; doi:10.3389/fpsyt.2018.00047)

Schizophrenia and Category-Selectivity in the Brain: Normal for Faces but Abnormal for Houses

Lisa Kronbichler^a,c*^, Renate Stelzig-Schöler^b^, Brandy-Gale Pearce^b^, Melanie Tschernegg^a^, Sarah Said-Yürekli^a,c^, Antonia-Luise Reich ^d^, Stefanie Weber^b^, Wolfgang Aichhorn^b^ & Martin Kronbichler^a,c^

^a^ Centre for Cognitive Neuroscience and Department of Psychology, University of Salzburg, Salzburg, Austria

^b^ Department of Psychiatry and Psychotherapy I, Christian-Doppler Medical Centre, Paracelsus Medical University, Salzburg, Austria

^c^ Neuroscience Institute, Christian-Doppler Medical Centre, Paracelsus Medical University, Salzburg, Austria

^d^ University Medical Center Hamburg-Eppendorf, Department of Psychiatry and Psychotherapy, Hamburg, Germany.

*Methods*

*fMRI Data Analysis*

*A priori ROIs*

ROIs included left FFA, right FFA, left PPA, right PPA, and for additional analyses: left and right STS and left and right OFA. The ROIS are freely available at http://www.brainactivityatlas.org). Data were acquired with a 3T Scanner in more than 200 participants during passive viewing of stimuli presented in a blocked design. Further characteristics of these ROIs are described in Zhen et al. (2015; 2017). Statistics were conducted in concatenated Session 1 and Session 2 of the fMRI.

*Individual ROIs*

In the individual ROI analysis, we searched within the a-priori ROIS for significant activation for each participant separately. In face-sensitive ROIs, all voxels exceeding a threshold of *p* <.01 (uncorrected) for the *face>scene* contrast of Session 1 were saved as an individual mask for each ROI and participant. The same process was applied to the scene-sensitive ROIs using the *scene>face* contrast. In a second step, we extracted the beta estimates of Session 2 of these individual ROIs. We were able to extract data from all participants within bilateral FFA and left PPA. One participant did not show significant voxels in the right PPA and was therefore reported as missing value in the analyses. Peak coordinates and voxel extent are reported in table SUP 1.


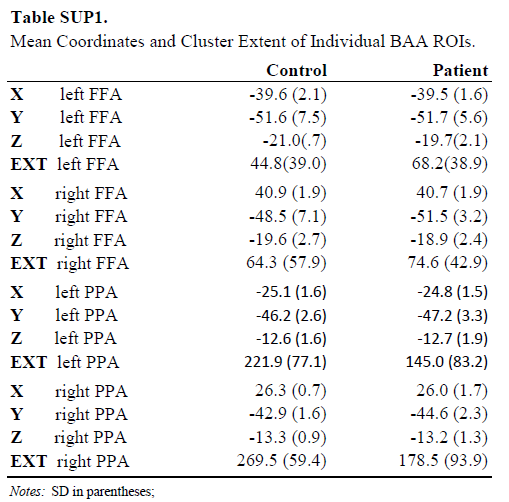


*Behavioral Results*

Eight healthy controls and nine patients with SZ must be excluded from behavioral analyses since they did not press any button during the whole experiment (although explicitly told to do so in the beginning). A repeated measures anova with one within subject factor (stimulus type) and one between subject factor (group) was designed. Irrespective of stimulus type, SZ patients revealed overall slower response times (*M* = 640.27, *SD* = 37.85) compared to healthy controls (*M* = 612.25, *SD* = 42.33) (*F*(1,35) = 4.43, *p* = .043). Both groups were slower at detecting inverted house targets compared to inverted face targets (*F*(1,35) = 23.62, *p* < .001).

Analyses of accuracy rates showed that participants were more accurate in detecting face compared to house targets (*F*(1,35) = 4.23, *p* = .047). There was no observable difference in accuracy rates between SZ patients and healthy controls (*F*(1,35) = .878, *p* = .35), and no Stimulus-by-Group interaction could be detected (*F*(1,35) = .025, *p* = .875).

*Results additional ROI analyses*

**Group** BAA ROIS - OFA and STS results

Left and right STS show increased neural response for face compared to house stimuli (*F*s(1,57) > 3.67, *p*s < .06). No main effect of group and no interaction was observed (*F*s(1,57) < 1.13, *p*s > .29).

There were no significant main effects and no interaction observable in the right OFA (*F*s(1,57) < 1.22, *p*s > .27). Left OFA, however, revealed a significant stimulus-by-group interaction (*F*(1,57) = 11.87, *p* = .001). Post hoc t-tests showed that this interaction was driven by a decreased neural response in patients towards house stimuli ( (*t* =2.65, *p*s = .01) whereas no such group difference was observed for face stimuli (*t* = 0.99, *p* = .35).

All results are depicted in Figure SUP1.


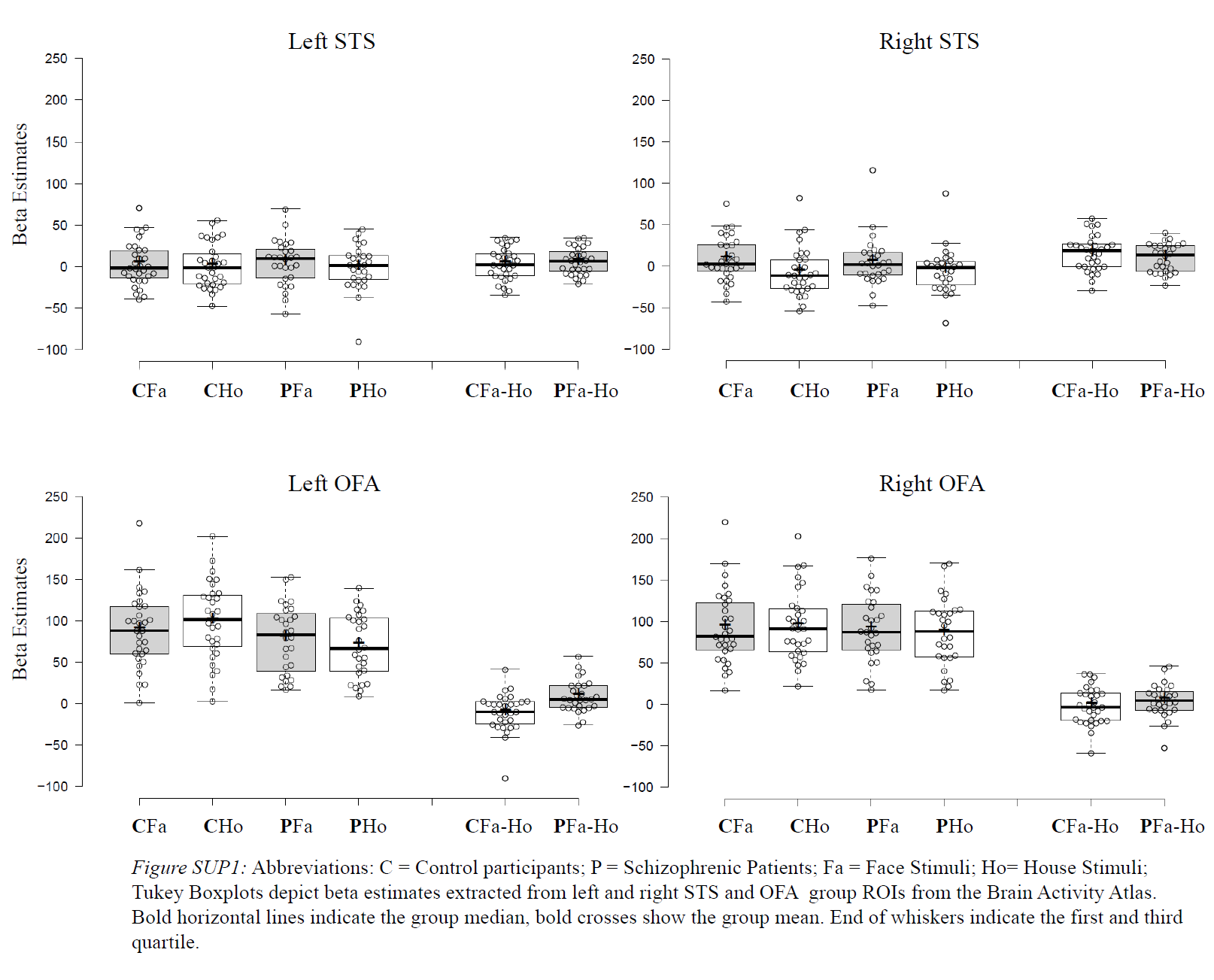


**Individually** defined BAA ROIS: PPA and FFA

In both FFA ROIs of the BAA atlas revealed significantly higher activation for face compared to house stimuli (*F*s(1,55) > 95.8, *p*s < .001). No main effect of group and no group-by-stimulus interaction could be observed (*F*s(1,55) < .04, *p*s > .84).

In left and right PPA ROI of the BAA atlas showed significantly higher activation for house compared to face stimuli (*F*s(1,55) > 160, *p*s < .001). This main effect was qualified by a significant stimulus-by-group interaction in right PPA (*F*(1,55) = 6.92, *p* = .011) and showed a similar tendency in left PPA (*F*(1,55) = 3.33, *p* = .073). There was no observable effect for group (*F*s(1,55) < .66, *p*s > .42). All results are depicted in Figure SUP2.


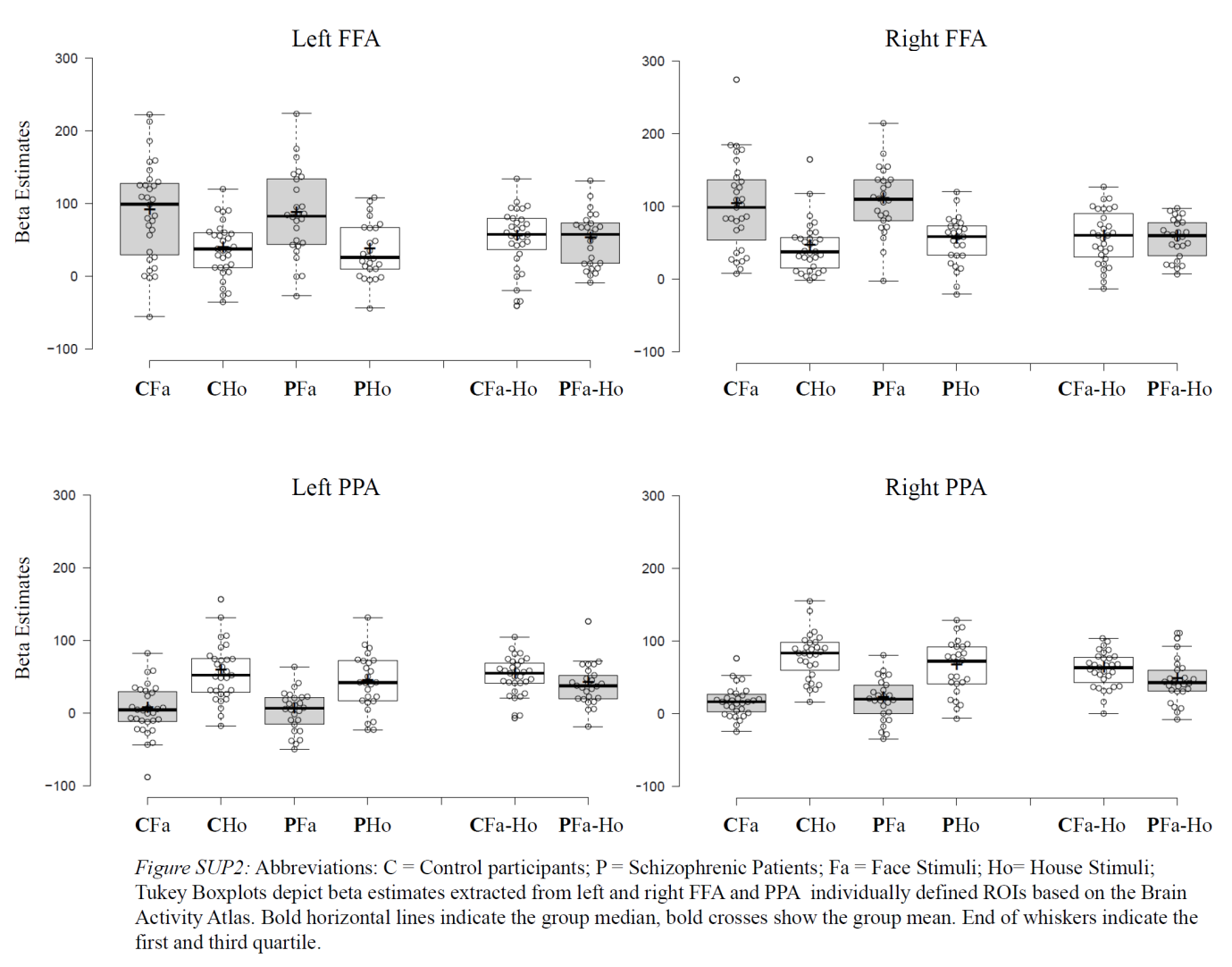


**Group** in-house ROIS

Left and right FFA ROI show significantly higher activation for face compared to house stimuli (*F*s(1,57) > 71.8, *p*s < .001). Overall activation in the left FFA was decreased for patients compared to controls (*F*(1,57) = 4.08, *p* = .048). However, there was no significant stimulus-by-group interaction in left and right FFA ROI (*F*s(1,57) < 1.99, *p*s > .16).

In both PPA ROIs, house stimuli revealed higher cortical response compared to face stimuli (*F*s(1,57) > 345.4, *p*s < .001) and patients showed generally decreased activation (*F*s(1,57) > 7.0, *p*s < .011). These main effects were qualified by a stimulus-by-group interaction in both ROIs (*F*s(1,57) > 19.6, *p*s < .001).


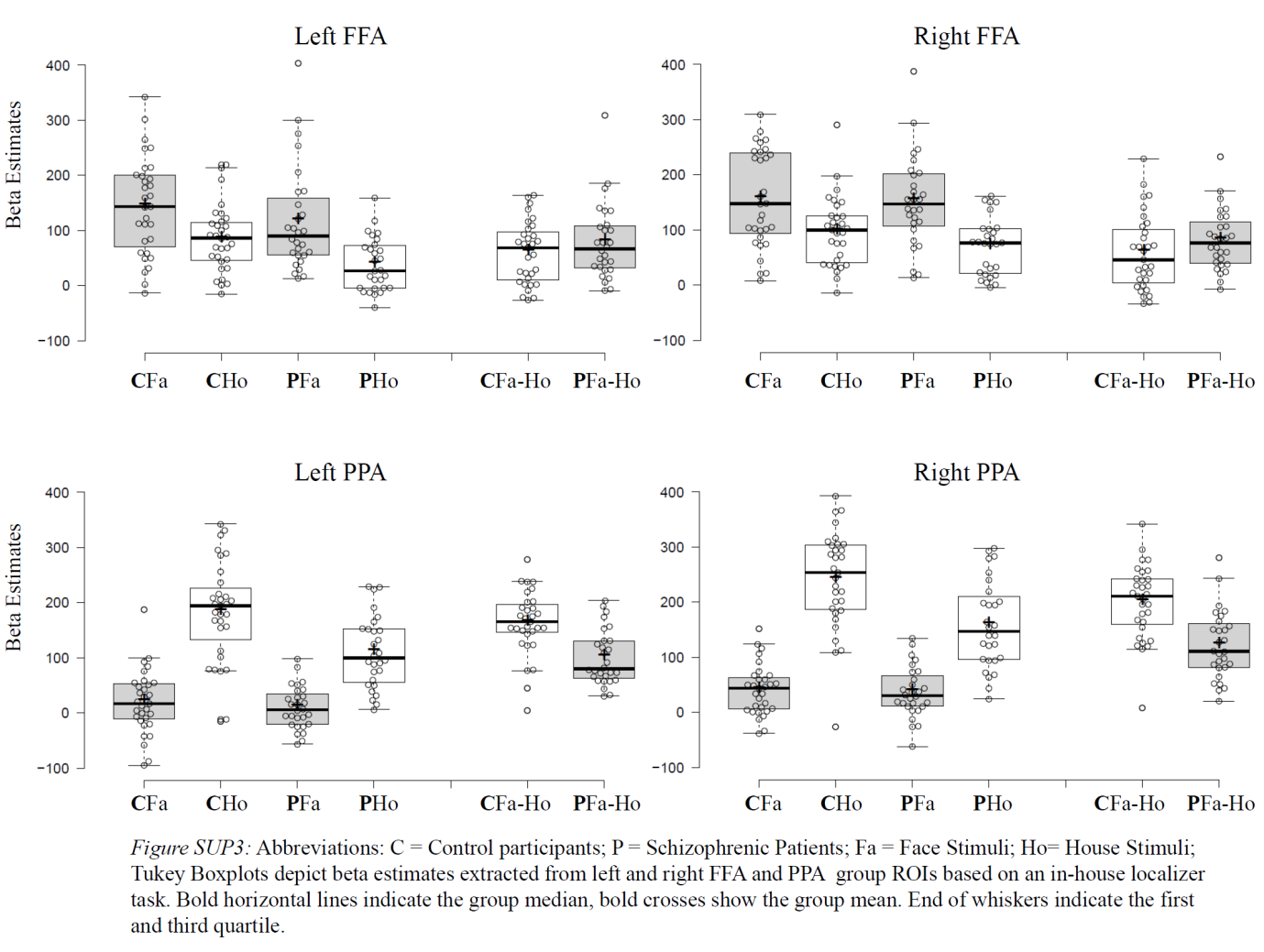


**Group** BAA ROIs with non-responsive participants (n=17) removed from analyses

Both FFA ROIs of the BAA atlas revealed significantly higher activation for face compared to house stimuli (*F*s(1,40) > 30.4, *p*s < .001). A stimulus-by-group interaction was only marginally significant in the left FFA (*F*(1,40) =3.8 , *p* = .058) and driven by decreased neural response of patients towards house stimuli.

Left and right PPA ROI of the BAA atlas showed significantly higher activation for house compared to face stimuli (*F*s(1,40) > 212.4, *p*s < .002). This main effect was qualified by a significant stimulus-by-group interaction in left and right PPA (*F*s(1,40) > 8.1, *p*s < .008). Post-hoc *t-tests* revealed significantly lower neural response of SZ patients (compared to controls) towards house stimuli (*t*s >2.3, *p*s < .025) but similar neural response for face stimuli (*t*s < .65, *p*s > .52) in both PPA clusters.

**Group** BAA fusiform face area ROI with the additional factor *Hemisphere*

An additional ANOVA with the factors stimulus (face / house), group (patients/controls) and hemisphere (left/right FFA) revealed a main effect of hemisphere, with generally increased activation in the right hemisphere (*F*(1,57) =9.15 , *p* = .004). However, no significant interaction with the factor hemisphere could be identified (*F*s(1,57) < 2.59, *p*s > .112).


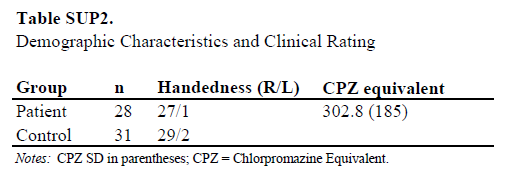


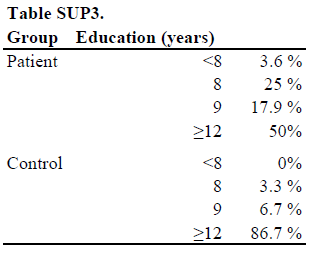


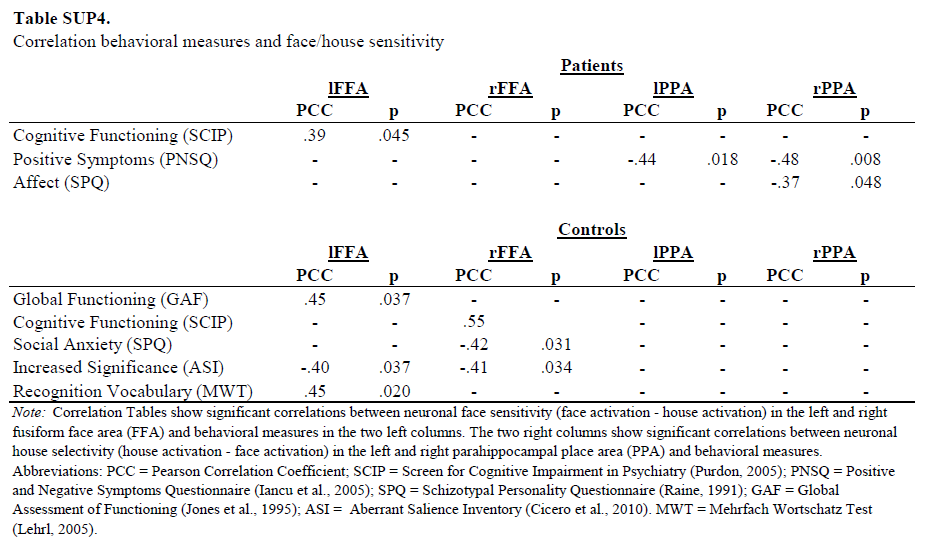

Supplement: Supplementary file 1 [file Data_Sheet_1.DOCX]
